# Data on docking and dynamics simulation of *Entamoeba histolytica* EhADH (an ALIX protein) and lysobisphosphatidic acid

**DOI:** 10.1016/j.dib.2016.02.067

**Published:** 2016-03-03

**Authors:** Silvia Castellanos-Castro, Sarita Montaño, Esther Orozco

**Affiliations:** a Departamento de Infectómica y Patogénesis Molecular, Centro de Investigación y de Estudios Avanzados del IPN Av. IPN 2508, San Pedro Zacatenco, México, D.F., 07360, México; b Colegio de Ciencia y Tecnología, Universidad Autónoma de la Ciudad de México, Dr. García Diego 168, CP 06720, México D.F., Mexico

**Keywords:** E. histolytica, LBPA, Molecular docking, Dynamics simulation

## Abstract

*Entamoeba histolytica* is the protozoan agent responsible for human amoebiasis. Trophozoites are highly phagocytic cells and the lysobisphosphatidic acid (LBPA) is involved in endocytosis. LBPA interacts with EhADH protein (an ALIX family member) also participating in phagocytosis, as it is referred in the research article *Identification of the phospholipid lysobisphosphatidic acid in the protozoan Entamoeba histolytica: an active molecule in endocytosis* (Castellanos-Castro et al., 2016) [1]. To unveil the interaction site between EhADH and LBPA, here we performed molecular modeling, dynamics simulation and docking. Molecular modeling and docking predictions revealed that EhADH interacts with LBPA through the Bro1 domain, located at the N-terminus of the protein and through the adherence domain at the C-terminus. In silico mutation abolished these interactions, supporting the data obtained in molecular dynamic and docking in silico assays.

**Specifications Table**

**Table:** 

Subject area	*Infectomics*
More specific subject area	*Bioinformatics and molecular dynamics simulation*
Type of data	*Table, figure*
How data was acquired	*NAMD software was used to perform a molecular dynamics simulation. For optimization of LBPA structure we used Gaussian 03 software and Molecular Operating Environment (MOE) for docking studies*
Data format	*Text format*
Experimental factors	*Amino acids sequence of EhADH was retrieved from UniprotKB database, crystal structure of ALIX was retrieved from Protein Data Bank*
Experimental features	*The docking was done at different snapshots of molecular dynamics simulation*
Data source location	*Data are within this article*
Data accessibility	*Data are supplied with this article*

**Value of the data**

• Docking analyses to predict the interacting sites between EhADH protein and phospholipid ligand LBPA.
• In silico mutation of EhADH interacting site with LBPA as an important tool to further support the docking predictions and perform the experiments with a higher precision.
• Molecular dynamic simulation of the mutated conformers to confirm in silico the specificity of interaction sites between EhADH and LBPA.

## 1 Data

Data presented here show: i) the in silico predicted interaction site between LBPA and EhADH (Supplementary Fig. S1), ii) the structural alignment of ALIX Bro1 domain sites that interact with LBPA (Supplementary Fig. S2) and iii) the docking of the mutated EhADH Bro1 domain and LBPA (Supplementary Fig. S3 and Table 1).

## 2 Experimental design, materials, and methods

### 2.1 Docking analysis of interaction between EhADH and LBPA

The 3D molecular model of EhADH was built with I-TASSER server [2] using the amino acid sequence Q9U7F6 (UniprotKB). Protein structure alignment was carried out with CHIMERA 1.10.1 software [3], using the Alix 2R03 sequence protein from PDB and EhADH 3D structure obtained with I-TASSER. For docking studies we used Molecular Operating Environment (MOE) (Chemical Computing Group Inc.) software, in an iMac 2.7 GHz Intel core I5. The interacting site between LBPA and EhADH was analyzed using the EhADH full-length sequence. Geometry optimization of 2,2′-bisoleoyl-LBPA ligand was done with Gaussian 03 software, utilizing AM1 base [4].

1. *In silico mutations were performed on residues indicated in* Table 1*.*
2. *Molecular dynamics simulation of mutated EhADH Bro1 domain.*

Molecular dynamics (MD) simulation of mutated Bro1 domain was performed on Cluster hybrid-Xiuhcoatl (http://clusterhibrido.cinvestav.mx/) using the NAMD 2.8 software [5] and CHARMM27 [6] force field in GPU-CUDA. Periodic boundary conditions were using in MD simulation. Particle MeshEwald (PME) was used to measure electrostatic interactions. Force field parameters for non-bonded cutoff, 9 Å and 2 fs time step were used. The hydrogen atoms were added using software psfgen from VMD program [6]. The system was submitted to minimization energy for 1000 steps followed by equilibration for 1 ns and simulation was continued without restrains. MD simulation ran was done for 25 ns using the NTV ensemble. After MD, docking was performed with LBPA at 0, 1 and 10 ns.

## Appendix A Supplementary material

Supplementary Fig. 1. In silico predicted interaction site between LBPA and EhADH. (A) Ribbon model of docking between EhADH and LBPA showing the binding sites at N and C-terminus, respectively. Red: 2,2´-bisoleoyl-LBPA. Green loops: interacting site located at the EhADH Bro1 domain corresponding to (B) 94-QEDSKPKK-101 and 224-FYKI-227. (C) Interacting site located at the EhADH C- terminus. Supplementary Fig. 2. Structural alignment of Alix and EhADH proteins. Alix Bro 1 domain in yellow (PDB: 2R03) and EhADH Bro1 domain in purple (UniprotKB: Q9U7F6). The loop in red corresponds to the LBPA interacting site. Supplementary Fig. 3. The docking analyses with the Bro1 domain mutated and LBPA after molecular dynamics simulations at (A): 0 ns, (B): 1 ns, (C): 10 ns. In red a phospholipid fragment that did not interact with the protein. Supplementary material
