# Supplementary material for: Data on docking and dynamics simulation of Entamoeba histolytica EhADH (an ALIX protein) and lysobisphosphatidic acid
Source: Data Brief. 2016 Mar 3;7:457–9. doi: 10.1016/j.dib.2016.02.067 (PMC4789338; doi:10.1016/j.dib.2016.02.067)
Supplement: Supplementary file 1 — Supplementary Fig. 1. In silico predicted interaction site between LBPA and EhADH. (A) Ribbon model of docking between EhADH and LBPA showing the binding sites at N and C-terminus, respectively. Red: 2,2´-bisoleoyl-LBPA. Green loops: interacting site located at the EhADH Bro1 domain corresponding to (B) 94-QEDSKPKK-101 and 224-FYKI-227. (C) Interacting site located at the EhADH C- terminus. Supplementary Fig. 2. Structural alignment of Alix and EhADH proteins. Alix Bro 1 domain in yellow (PDB: 2R03) and EhADH Bro1 domain in purple (UniprotKB: Q9U7F6). The loop in red corresponds to the LBPA interacting site. Supplementary Fig. 3. The docking analyses with the Bro1 domain mutated and LBPA after molecular dynamics simulations at (A): 0 ns, (B): 1 ns, (C): 10 ns. In red a phospholipid fragment that did not interact with the protein. Supplementary material [file mmc1.doc]

We have not conflict of interest of any type in carrying out the experiments and data presented here and nor in publishing our data that have not been published elsewhere.
