# Supplementary material for: Data on docking and dynamics simulation of Entamoeba histolytica EhADH (an ALIX protein) and lysobisphosphatidic acid
Source: Data Brief. 2016 Mar 3;7:457–9. doi: 10.1016/j.dib.2016.02.067 (PMC4789338; doi:10.1016/j.dib.2016.02.067)
Supplement: Supplementary file 2 — Supplementary material [file mmc2.zip › DIB-D15-00734/Table 1.docx]

Table 1. EhADH residues interacting with LBPA

*After mutations no binding between EhADH and LBPA in docking analysis was detected.

| **Bro1 domain** | | **V shape domain** | |  |
| --- | --- | --- | --- | --- |
| Residues interacting with LBPA  **(-5.8037 kcal/mol)** | Mutations* | Residues interacting with LBPA  **(-6.5384 kcal/mol)** | Mutations* | |
| D96 | - |  |  |  |
| S97 | S97A | E444 | E444A |  |
| K98 | K98A | E447 | E447A |  |
| P99 | - | Q651 | Q651A |  |
| K101 | K101A | N652 | N652I |  |
| Y225 | Deleted | Q655 | Q655A |  |
| I227 | Deleted | R663 | R663I |  |
