# Supplementary figures and images for: Data on docking and dynamics simulation of Entamoeba histolytica EhADH (an ALIX protein) and lysobisphosphatidic acid
Source: Data Brief. 2016 Mar 3;7:457–9. doi: 10.1016/j.dib.2016.02.067 (PMC4789338; doi:10.1016/j.dib.2016.02.067)

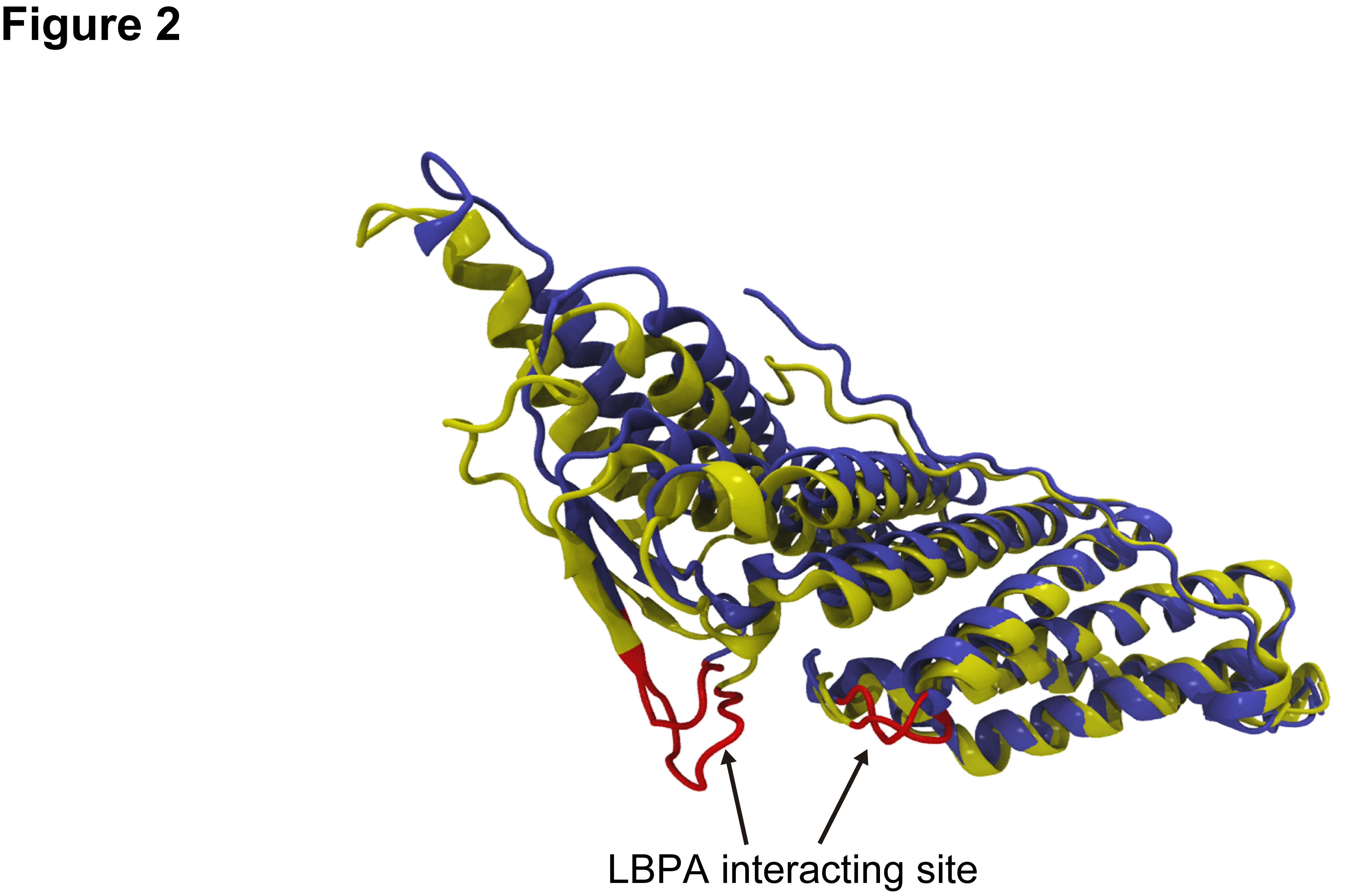

Supplement: Supplementary file 2 — Supplementary material [file mmc2.zip › DIB-D15-00734/Fig 2.tif]
